# Editorial Remarks

**Published:** 1843-01-21

**Authors:** 


### THE MEDICAL EXAMINER.

PHILADELPHIA, JANUARY 21, 1843.

After a lapse of nearly four years the present editor resumes his connection with this Journal, and in future it will be edited and conducted solely by himself. He trusts the opportunities he has enjoyed and the experience he has gained during that time will enable him to discharge his new duties satisfactorily. It will be his endeavour to maintain the practical character of the journal, which hitherto has contributed to render it so acceptable to the profession. The prominent and distinctive feature of the Examiner, will be the publication of Clinical Lectures and Hospital Reports. Arrangements have been effected by which permanent contributions of this character may be depended on for the current year. The reception of all the American and European medical periodicals, and of the new publications, place ample means in the hands of the editor, to enable him to present a faithful record of the progress of the medical sciences to the readers of the Examiner, and the short interval of publication offers the medium of a speedy communication of all the matters of interest. In addition to these ample foreign and domestic sources, the prominent clinical lectures delivered during the winter at Paris will be published immediately on their receipt, thus giving a faithful and interesting picture of the actual condition of medicine in the French metropolis. The valuable contributions of the numerous and respectable body of practitioners throughout the country, among whom this journal circulates, are particularly solicited. With these facilities, and with a determination of devoting his humble energies to the best interests of the entire profession, the editor solicits a continuation of the patronage which has hitherto been so liberally bestowed on this Journal.

